# Personality Development during Teacher Preparation

**DOI:** 10.3389/fpsyg.2016.01677

**Published:** 2016-11-08

**Authors:** Roisin P. Corcoran, Joanne O’Flaherty

**Affiliations:** ^1^School of Education, Johns Hopkins University, BaltimoreMD, USA; ^2^School of Education, University of LimerickLimerick, Ireland

**Keywords:** personality traits, IPIP Big-Five factor markers, latent growth curve analysis, academic attainment, social desirability

## Abstract

**Objective:** The purpose of this 3-year longitudinal study was to examine pre-service teachers’ personality trajectories as measured by the IPIP Big-Five factor markers during teacher preparation. The relationship between students’ personality traits, social desirability, and prior academic attainment was also examined.

**Method:** This 3-year longitudinal study invited participants from the first year of a 4-year undergraduate (UG) pre-service teacher education program, the class of 2017. The sample consisted of 305 students.

**Results:** The results suggest that extraversion, agreeableness, conscientiousness, emotional stability, and openness to experience were best represented by a non-significant longitudinal change in means. Results also suggest that social desirability predicts agreeableness and emotional stability with small to moderate effect sizes.

**Conclusion:** The study concludes that no value is added to pre-service teachers’ personality traits during 3 years of tertiary education. Furthermore, the data presented does not support the view that academic attainment is a good predictor of personality traits. Implications for educational research, theory, and practice are considered.

Decades of psychological research have found that an already well-established taxonomy of personality traits, called the Big Five factors, are a set of constructs that are most strongly predictive of valued societal outcomes across domains of life, school, and work (Schmidt and Hunter, 1998; MacCann et al., 2009; Poropat, 2009; Kyllonen et al., 2014). As a result, there is a growing body of literature looking at personality factors in the context of teaching, learning, and learning to teach (Adewale, 2013; Klassen and Tze, 2014; Spencer, 2014, unpublished). Entry into teacher education programs is often based on academic criteria alone with a view to admitting high quality candidates; however, critics argue that personal suitability data should accompany academic criteria as a way to recruit and retain more effective applicants into teaching positions (Corcoran and Tormey, 2010, 2012a,b, 2013; O’Flaherty and Gleeson, 2014; O’Flaherty and McGarr, 2014). In order to realize this, teacher preparation programs need to strengthen their understanding of the pre-teaching characteristics that significantly predict teacher performance and/or student outcomes (Corcoran and Tormey, 2013). There has been some focus on the sort of teacher education needed to support the development of teachers’ personality so that can develop these skills in young people (Bastian et al., 2015; Cheng and Zamarro, 2016). While there is evident value in such studies, there are also clear limitations. Recognizing that educators should develop these skills does not tell us how competent teachers and beginning teachers are in each of Big Five factors, and in which areas they most need to develop their skills. That is the issue which this paper addresses with respect to pre-service teacher education.

In the next section the Big Five personality factors are described, their importance with respect to teachers’ work is assessed and potential questions such as pre-service teachers’ personality development, the relationships between prior academic achievement and personality and between social desirability and personality are identified. Thereafter the methodology used in the research is described and then the findings from a longitudinal study on personality in pre-service teacher education students are reported. The discussion section identifies which personality factors are most and least problematic for the pre-service teachers studied and which need to be addressed in their teacher education programs.

## Conceptual Framework

### Personality

Allport’s (1938) text, *Personality: A Psychological Interpretation*, attempts to define and systematize the field of personality psychology. In it he defines personality as “the dynamic organization within the individual of those psychophysical systems that determine his unique adjustments to his environment.” While there is a range of different models for personality, there is some degree of consensus that the Big Five personality factors (neuroticism, extraversion, openness, agreeableness, and conscientiousness) capture most of the individual differences in behavioral patterns (Tupes and Christal, 1961; Costa and McCrae, 1985), and are therefore appropriate for studying daily behavior and performance in a wide range of domains. Collectively, they are known as the Five-Factor Model (FFM) of personality or the ‘Big Five’ and are considered assessable via personality inventories (Goldberg et al., 2006). The FFM emerged from a factor analysis of people’s ratings on the extent to which traits listed in the dictionary were descriptive of them. The idea of using dictionary terms for personality traits follows from the “lexical hypothesis,” or the idea that all of the most important personality characteristics of people should be encoded into their language (Kyllonen et al., 2014). The factors of the FFM of personality are extraversion, agreeableness, conscientiousness, emotional stability, and openness to experience. Among the Big Five personality traits, conscientiousness and neuroticism are the best predictors of work related performance (Salgado, 1997). In an educational context, numerous studies have explored the relationship between the Big Five, especially the trait of conscientiousness, and academic achievement (AA). Across various educational settings, personality traits have been shown to contribute to the explanation of individual differences in AA (Noftle and Robins, 2007; Chamorro-Premuzic and Furnham, 2008; Furnham and Monsen, 2009; Poropat, 2009; De Feyter et al., 2012). Studies on high-school students (Heaven et al., 2007; Laidra et al., 2007; Lesson et al., 2008) and college students (Chamorro-Premuzic and Furnham, 2003a,b; Furnham et al., 2003) discuss the relationship between personality and academic performance. There has also been some focus on whether or not the personality traits of pre-service teachers impacts teaching performance (Arif et al., 2012; Adewale, 2013; Klassen and Tze, 2014; Spencer, 2014, unpublished).

Previous studies have suggested that personality development is ‘relatively stable, enduring and important aspects of the self’ (Maltby et al., 2007, p. 9). Personality trait theorists and some studies of rank-order stability suggest personality attributes are stable and are more or less domain-general meaning the same person is likely to have similar attributes over time, and they are enduring because the same person will have similar attributes in different social contexts. Possible reasons for such stability in personality include genetic influences, environmental influences (e.g., stability in personality is as a result of a stable environment) and person-environment transactions (e.g., people seek information and experiences congruent with their self-image). However, from a developmental standpoint personality can and does change over time and context, and is important in understanding people’s actions. This highlights a major controversy related to personality theories, that is, the degree to which personality can or does change. More recent methods for assessing the continuity and change of personality traits—including mean-level change, individual-level change, and ipsative continuity—indicate that personality traits are malleable. Mean-level change studies show that while young adulthood is the period for the most significant changes, personality does continue to develop throughout adulthood and even into late adulthood (Roberts and DelVecchio, 2000; Roberts et al., 2006). Normative changes move in a positive direction with people becoming more conscientious and emotionally stable as they age. Studies of individual-level change show that most people remain stable. Of those who do exhibit change, most exhibit normative development, but there are individuals who develop in a non-normative manner. Ipsative continuity studies suggest that there is moderate-high profile similarity during childhood and adolescence. Possible reasons for change in personality include norms about appropriateness of behaviors and goals at different ages in different cultures, change in roles, social learning, expectable versus unexpected life-changes, and a significant life event. Block (1971) suggests that personality changes as a result of maturation, societal pressure, “biosocial norm” and sometimes the person appears different because norms change. For example, Robins et al. (2001) measured the Big Five personality traits in college students at the beginning and the end of college and concluded that going to college can change students’ personality and promote desirable adult development. These findings, particularly the conclusion that personality is not set in stone, but instead continues to develop into adulthood, have implications for many important outcomes. However, more longitudinal studies are needed and no studies exist which examine the trajectories of pre-service teachers’ personality traits. For that reason, it is important to investigate personality traits development among pre-service teachers and predictors of change in personality traits using LGM, which provides more rigorous examinations to detect heterogeneity in change across participants.

### Personality and Teachers’ Work

The importance of teacher personality has long been of interest to education researchers (e.g., Tyler, 1960; Barr, 1961). While associations can differ depending on various factors related to how personality is conceptualized and measured (i.e., the bandwidth-fidelity debate; self-rated versus observer-rated), considerable research has been conducted based on the assumption that teacher personality domains are associated with teaching effectiveness. While evidence for this assumption has a controversial history (Klassen and Tze, 2014), there is some evidence supporting the view (Adewale, 2013; Spencer, 2014, unpublished). Tonelson’s (1981) seminal work shows a relationship between teacher personality and learning atmosphere suggesting that teacher personality can effect student learning outcomes through the psychological environment of the class. Kagan and Grandgenett (1987) in their review of the literature noted a consistent relationship between teacher personality traits and preferred instructional style, while Lorentz and Coker (1977) reported a significant relationship between teachers’ scores on the Myers-Briggs Type Indicator (MBTI) and the behavior of their students, concluding that the teachers’ personality impacted upon student behavior in the classroom. Gordon and Yocke (1999) assessed the relationship between personality types, as measured by the MBTI Form G, and teaching effectiveness using the Classroom Observations Keyed for Effectiveness Research (COKER). Results indicate that whilst the majority of respondents reported a preference for ‘extraversion sensing-thinking-judging (ESTJ),’ the sample scored well below the mean on the 18 COKER competency statements, with only 41% being classified as ‘effective teachers.’ More recently, the importance of measuring teacher conscientiousness has been of rising research interest as studies indicate that teacher conscientiousness predicts student educational outcomes. Klassen and Tze (2014) completed a meta-analysis of 43 studies involving 9,216 teachers and reported that teachers’ personality is significantly and positively related to teaching performance (*r* = 0.10). While Murray (1972) suggests that personality influences the behavior of the teacher in various ways such as engagement and relationships with students, pedagogical approaches, and learning experiences selected. However, only a few longitudinal studies have addressed the relationship between personality and relationship change in adolescence and early adulthood (Neyer and Asendorpf, 2001; Robins et al., 2002; Asendorpf and van Aken, 2003; Branje et al., 2004; Neyer and Lehnart, 2007). The consistent picture emerging from these studies is that personality effects on change in relationships are more powerful and more frequent than relationship effects on personality change.

Conscientiousness refers to an individual’s propensity for planning, organizing, carrying out tasks, and for being reliable, purposeful, strong-willed and determined (Costa and McCrae, 2006). The importance of measuring teacher conscientiousness has been of rising research interest as studies indicate that teacher conscientiousness predicts student educational outcomes. Nguyen et al. (2005) found that individuals who exhibit higher levels of conscientiousness on FFM measures are more likely to transfer their learning within educative environments. Cheng and Zamarro (2016) report that measures of teacher conscientiousness capture important dimensions of teacher quality, for example, teachers with higher conscientious scores are more effective at improving their student conscientiousness. While Bastian et al. (2015) equally support the focus on investigation of beginning teacher personality traits and report that conscientiousness significantly predicts higher value-added, higher evaluation ratings, and higher rates of retention of teachers in North Carolina public schools. Job (2004) describes a weak positive correlation between conscientiousness and teaching effectiveness (*r* = 0.03). Chiang (1991) report modest relationships between the ‘feeling –thinking’ branch of the MBTI and the following measures of teacher performance: organization (*r* = -0.17); teacher–student rapport (*r* = -0.20); teaching skill (*r* = -0.37); global rating of teacher (*r* = -0.29); and class management (*r* = -0.22). While Rockoff et al. (2008) describe a weak positive correlation between conscientiousness and students’ math scores (*r* = 0.01). Stewart et al. (2008) report positive correlations between the application of coaching development and conscientiousness, openness to experience, emotional stability and general self-efficacy. More importantly conscientiousness was found to be associated with maintenance of coaching outcomes.

Within other workplace domains, of the five main personality factors, conscientiousness has been shown to be the most consistent, significant predictor of workplace performance (Barrick and Mount, 1991; Behling, 1998; Hogan and Holland, 2003; Dudley et al., 2006). Meta-analyses conducted by Hurtz and Donovan (2000) and Barrick et al. (2001) have demonstrated that measures of conscientiousness predict overall job performance (Hough and Oswald, 2008) including the prediction of valued workplace behaviors, such as leadership (Judge et al., 2002) as well as undesirable behaviors such as procrastination (Judge and Ilies, 2002).

Openness refers to an individual’s curiosity about their inner and outer worlds, their willingness to entertain novel ideas and unconventional values, and the intensity with which they experience their emotions (Costa and McCrae, 2006). Bastian et al. (2015) report that first-year teachers with higher levels of openness to experience were significantly more likely to work in high-need school environments (that is high-poverty, high-minority, and low performing schools). Job (2004) describes a weak negative correlation between openness scores and teaching effectiveness (*r* = -0.04). Barrick and Mount (1991) found that openness was positively related to performance for managers and negatively related to performance for professionals.

Emotional stability refers to an individual’s tendency toward being calm, even-tempered and relaxed, and their ability to face stressful situations without upset (Costa and McCrae, 2006). Studies in the area have linked teacher effectiveness with emotional stability (Gage, 1965). Furthermore, the emotional stability and skills of teachers’ influences student conduct, engagement, attachment to school, and academic performance (Baker, 1999; Hawkins et al., 1999; Wentzel, 2002; Durlak et al., 2011). Jamil et al. (2012) report small correlations between neuroticism scores and the following teacher performance assessments: emotional support (*r* = 0.09); classroom organization (*r* = -0.02); instructional support (*r* = 0.04). Job (2004) describes a weak negative correlation between neuroticism scores and teaching effectiveness (*r* = -0.10). Chiang (1991) report modest relationships between neuroticism and the following measures of teacher performance: teaching skill (*r* = 0.19); student participation (*r* = -0.013); and class management (*r* = 0.13).

Agreeableness refers to an individual’s tendency toward being friendly and compassionate as opposed to analytical and detached (Costa and McCrae, 2006). Bastian et al. (2015) report that agreeableness is negatively associated with teacher EVAAS estimates (the official measure of teacher effectiveness for teacher evaluation in North Carolina). While Chu (2003) reports modest correlations between agreeableness and principal evaluation (*r* = 0.12). Job (2004) describes a weak negative correlation between agreeableness and teaching effectiveness (*r* = -0.04). Mkoji and Sikalieh (2012) suggest that agreeable individuals who possess the ability to adapt in the workplace display increased performance. Agreeableness has been shown to predict performance in interpersonal-oriented jobs (Hurtz and Donovan, 2000).

Extraversion refers to an individual’s tendency toward being outgoing and energetic versus solitary and reserved (Costa and McCrae, 2006). Earlier studies in the area have linked teacher effectiveness with extraversion (Srivastava and Bhargava, 1984). Chan (2003) report a moderate negative correlation between extraversion scores and teaching effectiveness assessment (*r* = -0.40). Chiang (1991) report modest positive relationships (*r* = 0.18–0.37) between extraversion and the following measures of teacher performance: teaching skill (0.18); class management (0.23); organization (0.20). Jamil et al. (2012) report small correlations between extraversion scores and the following teacher performance assessments: emotional support (*r* = 0.03); classroom organization (*r* = 0.01); instructional support (*r* = 0.02). Kent and Fisher (1997, p. 8) report that teachers who displayed higher scores of extraversion perceived their classrooms as characterized by high levels of ‘student cohesion (the extent to which students know, help and are friendly toward each other)’. Fisher et al. (1998) investigated the relationship between student and teacher perception of teacher–student interpersonal behavior using the Questionnaire on Teacher Interaction (QTI) and teacher personality using the MBTI. Results indicated that teachers’ personality was related to student perceptions of their teachers’ interpersonal behavior, particularly in terms of how much freedom or responsibility students perceived they were given. While Rockoff et al. (2008) describe a weak positive correlation between extraversion and students’ math scores (*r* = 0.01). Extraversion has been positively correlated with occupations that require social interactions (Barrick and Mount, 1991) and leadership abilities (Lim and Ployhart, 2004). Extraverts tend to search for social relationships with co-workers (Louis et al., 1983) which is associated with positive workplace performance (Mkoji and Sikalieh, 2012).

In summary, there is a growing body of literature on various aspects of teacher personality. There is evidence that various personality traits are important for teaching and learning to teach, but there is limited evidence about the levels and trajectories of personality that pre-service teachers have. It is also unclear as to whether or not pre-service teachers’ personality development is associated with the route of entry into pre-service teacher education. The IPIP provides a conceptual framework for making sense of what specific levels of personality traits pre-service teachers have, and what are the trajectories of personality traits during this developmental period. This in turn has implications for program and intervention development.

## Method

This study sought to address the following questions:

(1) Are there changes in pre-service teachers’ personality factors (extraversion, agreeableness, conscientiousness, emotional stability, and openness to experience) during pre-service teacher education?
(2) Is there evidence of a relationship between students’ personality factors and prior academic achievement?
(3) Is there evidence of a relationship between students’ personality traits and social desirability?

### Research Context

This study was undertaken with secondary level teacher education students in Ireland. A complete evaluation of the research context is provided elsewhere (Corcoran and Tormey, 2012b); however, it should be noted that the Organization for Economic Cooperation and Development (OECD) has pointed to the competitiveness for entry and high social status traditionally enjoyed by the teaching career in Finland, Korea and Ireland (Organization for Economic Cooperation and Development [OECD], 2005). The number of places on offer each year is set by the Department of Education and Skills (DES). The undergraduate (UG) pre-service teachers that participated in the study had gained access to their program largely by academic grades on the state run, school-leaving examination (the Leaving Certificate). Teacher preparation programs generally comprise three components: foundation studies, pedagogy/methods and field experience. The Teaching Council of Ireland sets out that ‘programs should be designed in a demonstrably integrated way, incorporating foundation studies, professional studies, school placement and, as appropriate, subject disciplines’ (Teaching Council of Ireland, 2011, p. 10). Internationally, entry routes to pre-service teacher education programs are a frequent topic for debate (Cochran-Smith and Fries, 2005; Organization for Economic Cooperation and Development [OECD], 2005). The question as to whether a personality assessment tool like the IPIP might contribute some valuable information in a selection process is clearly one that holds interest in many countries.

### Participants

Students, from the first year of a 4-year UG pre-service teacher education program in an Irish university, were invited to participate in this study aimed at investigating longitudinal personality trait development. All students who agreed to participate in the research were selected. In the fall of 2013, the IPIP was administered to the participants (time 1), the class of 2017. So that participants could serve as their own controls and provide additional information about the changes that occurred during college education, we administered this same measure in the fall of the following year (2014; time 2) and again 1 year later in fall (2015; time 3). This sample consisted of 305 students, 185 (60.7%) males and 113 (37.0%) females. The students in question were studying a range of academic disciplines including: physical education (*n* = 65, 21.4%); biological science (*n* = 64, 21.0%), architectural technology (*n* = 57, 18.7%), engineering technology (*n* = 42, 13.8%), physical science (*n* = 15, 4.9%), and other studies (*n* = 12, 3.9%). Based on the information available, ages at baseline ranged from 17 to 53 years (*M* = 19.58, *SD* = 3.69). Ethical approval was sought and granted from the University Research Ethics Committee. Informed consent was obtained from all parties involved.

### Measures

#### Personality Traits

The Big Five dimensions of personality were assessed using Goldberg’s IPIP 50-item measure (Goldberg, 1999), which is widely regarded as both reliable and valid (Gow et al., 2005; Guenole, 2005; Mlačić and Goldberg, 2007). The Big Five personality factors are composed of five subscale scores: extraversion, agreeableness, conscientiousness, emotional stability, and openness to experience. The measure comprises of short sentences describing various behaviors associated with each of the Big Five dimensions of personality. Each scale contains 10 items paired with a 5-point Likert response rate (1 = strongly disagree, 2 = disagree, 3 = neutral, 4 = agree and 5 = strongly agree).

#### Social Desirability

One limitation of Goldberg’s scales is that it relies on self-reported assessments of personality. As with all self-report instruments, students may respond in ways that are socially desirable rather than reveal their honest response to each statement. To assess the extent to which participant may adapt their responses to present themselves in a favorable light, the 10-item valid and reliable short-form of the Marlowe-Crowne Social Desirability scale was administered (Crowne and Marlowe, 1960; Strahan and Gerbasi, 1972). Respondents indicate whether each of the items is true or false for them (sample item: “I never hesitate to go out of my way to help someone in trouble.”). Respondents receive a point for each response that “matches” the score key (for sample item, the match is *True*), and 0 for each response that does not match the score key. Higher scores reflect greater social desirability.

#### Academic Achievement Measure

Prior AA was measured by performance on the ‘Leaving Certificate,’ a state-organized, compulsory examination taken by students at the end of their second-level schooling (state equivalent of SAT scores). This assessment is a high-stakes test which determines entry into further education for the vast majority of students. Gormley and Murphy (2006) provide a further description of some features of the Leaving Certificate and point system.

### Analytic Issues

#### Latent Growth Curve Modeling

The development of personality traits was modeled using latent growth curve modeling or LGM. This technique is more flexible than traditional approaches, and more importantly, allows for the simultaneous modeling of multiple predictors. Models were fit using Mplus 7.31 (Muthen and Muthen, 2011). Model estimation was based on the robust maximum likelihood estimator (MLR; Yuan and Bentler, 2000). In LGM, growth parameters that describe the pattern of change for each construct are the intercept and slope, which are modeled as latent variables. Both of these latent variables have an associated mean and variance in these models. The intercept represents individual differences in the level of a particular construct at a particular time (e.g., initial status). The slope represents the linear trend of an individual’s trajectory across repeated measurements. The parameters of primary interest are the mean intercept and mean slope, which can be interpreted as the average level and slope of the trajectory across the sample. Factor loadings linking the intercept factor to the observed variables were set to 1.0 and loadings linking the linear factor to the observed variables represent time between the first administration of the IPIP scales and each subsequent wave of data collection. Hence, the factor loadings for the linear slope were 0, 1, and 2. These factor loadings represent the amount of time that passed in the intervening period between time points condensed to a similar metric. Time point one is always considered zero in growth models if the intercept is defined at the first wave of measurement and because the time metric is measured in years, time point two was 1 because data was collected a year later, and the final time point three was 2 because it was 1 year later from time point two. Correlations between the residuals of the observed variables that were measured on several occasions for the same subjects were allowed (Marsh and Hau, 1996).

Because LGM is an extension of structural equation modeling (SEM) procedures, the same goodness-of-fit criteria are applicable, and successively nested models can be evaluated against each other. In our LGMs, all constructs were measured with a single indicator and were therefore included as manifest (observed) variables in all models. Based on previous recommendations (Cole, 1987; Marsh et al., 1988), the indices selected to assess goodness-of-fit were as follows: the Root Mean Squared Error of Approximation (RMSEA), the Tucker-Lewis Index (TLI), and the Comparative Fit Index (CFI), as operationalized in Mplus in association with the MLR estimator. We also considered the robust χ^2^ test statistic and parameter estimates. The criteria used to indicate good fit, based on several evaluations (Anderson and Gerbing, 1984; Cole, 1987; Marsh et al., 1988; McDonald and Marsh, 1990), include the following: CFI > 0.9, TLI > 0.85, and RMSEA < 0.08.

## Results

### Preliminary Analyses

#### Sample Attrition

Attrition patterns for students were examined. For all personality scales, of the 305 students in the sample there were 162 (53.1%) students at time 1, 232 (76.1%) students in the study at the end of time 2, and 142 (46.5%) students remained in the study at the end of time 3. There is clearly a participant attrition rate at time 1 and time 3 in the sample. However, most of the participants rejoin the sample in time 2. Therefore, full information likelihood procedures were used to retain power in the analysis (Enders, 2001; Graham, 2009).

### Descriptive Statistics

Descriptive statistics are presented in **Table 1**. In the present study, the mean of extraversion at time 1 was 3.40 (*SD* = 0.63), at time 2 was 3.37 (*SD* = 0.60), and at time 3 was 3.27 (*SD* = 0.59). The mean of agreeableness at time 1 was 3.86 (*SD* = 0.53), at time 2 was 3.82 (*SD* = 0.53), and at time 3 was 3.75 (*SD* = 0.52). The mean of conscientiousness at time 1 was 3.42 (*SD* = 0.61), at time 2 was 3.41 (*SD* = 0.57), and at time 3 was 3.43 (*SD* = 0.55). The mean of emotional stability at time 1 was 3.10 (*SD* = 0.70), at time 2 was 3.17 (*SD* = 0.63), and at time 3 was 3.06 (*SD* = 0.73). The mean of openness at time 1 was 3.26 (*SD* = 0.55), at time 2 was 3.37 (*SD* = 0.51), and at time 3 was 3.31 (*SD* = 0.59). The mean of social desirability at time 1 was 5.61 (*SD* = 1.85). Our sample of pre-service teachers had a wide range of assessed prior academic achievement (range = 325 points-615 points; *M* = 471.08, *SD* = 51.106), and points were not available for 68 teachers (22%).

**Table 1:** Descriptive statistics of the Big Five personality traits.

	*M*	*SD*	α	ICC
**Extraversion**				
Time 1	3.4	0.63	0.812	0.302
Time 2	3.37	0.60	0.819	0.311
Time 3	3.27	0.59	0.795	0.280
**Agreeableness**				
Time 1	3.86	0.53	0.750	0.231
Time 2	3.82	0.53	0.777	0.259
Time 3	3.75	0.52	0.769	0.250
**Conscientiousness**				
Time 1	3.42	0.61	0.766	0.247
Time 2	3.41	0.57	0.762	0.242
Time 3	3.43	0.55	0.737	0.219
**Emotional Stability**				
Time 1	3.10	0.55	0.742	0.223
Time 2	3.17	0.51	0.727	0.210
Time 3	3.06	0.59	0.756	0.237
**Openness**				
Time 1	3.26	0.55	0.742	0.223
Time 2	3.37	0.51	0.727	0.210
Time 3	3.31	0.59	0.756	0.237

### Correlations between Measures

Spearman’s rho correlations were conducted for the variables used in the study. A correlation matrix is provided in **Table 2**. An inspection of the correlation coefficients revealed that social desirability was positively and significantly associated with agreeableness (*r* = 0.394, *p* < 0.01), and emotional stability (*r* = 0.246, *p* < 0.01). Interestingly, AA was not associated with any of the personality factor variables.

**Table 2:** Correlations among study variables.

Variable	AA	SDes	Extraversion	Agree	Conscientiousness	Stability	Open
AA	1.00
SDes	-0.060	1.00
E	0.074	0.034	1.00
A	0.033	0.394**	0.349**	1.00
C	0.067	0.083	-0.022	0.198*	1.00
ES	0.009	0.246**	0.167*	0.103	-0.003	1.00
OE	0.020	0.043	0.308**	0.330**	0.216**	0.151	1.00

*^∗^*p* < 0.05, ^∗∗^*p* < 0.01.*

### Modeling Growth Curves for Personality Traits Measures

#### Extraversion Measures

A linear model of the growth curve for extraversion scores resulted in an excellent fit [χ*^2^*(3) = 1.168, *p* = 0.761, CFI = 1.00, TLI = 1.02, RMSEA < 0.001]. The model accounts for 62–68% of the variance in the observed extraversion variables at the three times of measurement. Results indicated that the means at time 0 were greater than 0 (intercept: *M* = 3.377, *p* < 0.001), and that there was no increase in the mean change of extraversion over time (linear slope: *M* = -0.040, *p* = 0.077). There was significant variability in the initial values (intercept: *Var* = 0.274, *p* < 0.001), but not with the slope. In other words, there was significant inter-individual differences in initial levels of extraversion scores but not in growth trajectories. A non-significant correlation between the intercept and the linear slope indicates that participants with higher initial values did not significantly show more decrease over time compared to participants with lower initial values.

#### Agreeableness Measures

A linear model of the growth curve for agreeableness scores resulted in an excellent fit (χ*^2^*(3) = 4.079, *p* = 0.253, CFI = 0.974, TLI = 0.974, RMSEA = 0.036). The model accounts for 49–57% of the variance in the observed agreeableness variables at the three times of measurement. Results indicated that the means at time 0 were greater than 0 (intercept: *M* = 3.836, *p* < 0.001), while there was no increase in the mean change of agreeableness over time (linear slope: *M* = -0.030, *p* = 0.271). There was significant variability in the initial values (intercept: *Var* = 0.171, *p* < 0.001), and with the slope (slope: *Var* = 0.041, *p* = 0.017). In other words, there was significant inter-individual differences in initial levels and in growth trajectories of agreeableness scores. A significant correlation (*r* = -0.51, *p* < 0.001) between the intercept and the linear slope indicates that participants with higher initial values did significantly show more decrease over time compared to participants with lower initial values.

#### Conscientiousness Measures

A linear model of the growth curve for conscientiousness scores resulted in an excellent fit [χ*^2^*(3) = 1.122, *p* = 0.772, CFI = 1.00, TLI = 1.02, RMSEA < 0.001]. The model accounts for 66–72% of the variance in the observed conscientiousness variables at the three times of measurement. Results indicated that the means at time 0 were greater than 0 (intercept: *M* = 3.375, *p* < 0.001), while there was no increase in the mean change of conscientiousness over time (linear slope: *M* = 0.016, *p* = 0.533). There was significant variability in the initial values (intercept: *Var* = 0.278, *p* < 0.001), and with the slope (slope: *Var* = 0.035, *p* = 0.021). In other words, there was significant inter-individual differences in initial levels and in growth trajectories of conscientiousness scores. A significant correlation (*r* = -0.538, *p* < 0.001) between the intercept and the linear slope indicates that participants with higher initial values did significantly show more decrease over time compared to participants with lower initial values.

#### Emotional Stability Measures

A linear model of the growth curve for emotional stability scores resulted in an excellent fit [χ*^2^*(3) = 4.234, *p* = 0.237, CFI = 0.987, TLI = 0.987, RMSEA = 0.039]. The model accounts for 65–68% of the variance in the observed emotional stability variables at the three times of measurement. Results indicated that means at time 0 were greater than 0 (intercept: *M* = 3.155, *p* < 0.001), while there was no increase in the mean change of emotional stability over time (linear slope: *M* = -0.039, *p* = 0.184). There was significant variability in the initial values (intercept: *Var* = 0.309, *p* < 0.001), but not with the slope. In other words, there was significant inter-individual differences in initial levels of emotional stability scores but not in growth trajectories. A non-significant correlation (*r* = -0.254, *p* = 0.218) between the intercept and the linear slope indicates that participants with higher initial values did not significantly show more decrease over time compared to participants with lower initial values.

#### Openness Measures

A linear model of the growth curve for openness scores resulted in a weak fit [χ^2^(3) = 11.12, *p* = 0.011, CFI = 0.930, TLI = 0.930, RMSEA = 0.100]. The model accounts for 67–72% of the variance in the observed openness variables at the three times of measurement. Results indicated that the means at time 0 were greater than 0 (intercept: *M* = 3.284, *p* < 0.001), while there was no increase in the mean change of openness over time (linear slope: *M* = 0.035, *p* = 0.125). There was significant variability in the initial values (intercept: *Var* = 0.197, *p* < 0.001), and with the slope (slope: *Var* = 0.025, *p* < 0.047). In other words, there was significant inter-individual differences in initial levels of openness scores and in growth trajectories. A non-significant correlation (*r* = -0.242, *p* = 0.143) between the intercept and the linear slope indicates that participants with lower initial values did not significantly show more increase over time compared to participants with higher initial values.

## Discussion and Conclusion

Our first question asked is there changes in per-service teachers’ personality factors (extraversion, agreeableness, conscientiousness, emotional stability, and openness to experience) during pre-service teacher education? The findings show there was no increase in the mean change of extraversion, agreeableness, conscientiousness, emotional stability, and openness to experience during 3 years of tertiary education. This is an important finding in that is suggests that no value is added to pre-service teachers’ extraversion, agreeableness, conscientiousness, emotional stability, and openness to experience during 3 years of tertiary education. The personality trait pre-service teachers’ reported highest across all 3 years was agreeableness. Interestingly, the personality trait pre-service teachers’ reported lowest across all 3 years was emotional stability. As was noted above, the personality of teachers have been found to influence student academic performance (Bastian et al., 2015). Downey et al. (2014) studied the role of personality in academic success and report that that academic success was related to higher levels of emotional management control (EMC), conscientiousness and lower levels of extraversion thus emulating previous research that shows the role of emotional competencies in academic success. Pre-service teachers diminished growth, therefore is a cause for some concern. Plausible explanations for diminished growth include reduced motivation (Martin, 2007; Metallidou and Vlachou, 2010); perceived value (Metallidou and Vlachou, 2010) self-concept (King and McInerney, 2014), enjoyment, and self-efficacy beliefs (Pinxten et al., 2014) and engagement (Pascarella et al., 2004) during the college transition. According to Shin et al. (2013), another plausible reason for diminished growth is that third level education environments are more demanding than secondary school, and so sustained growth requires more effort. Many countries including the United States have experienced problems attracting and retaining effective teachers. Developing an understanding of the psychological profiles of pre-service teachers may help to better prepare teachers for the demands of the profession—and ultimately attract, support and retain effective teachers.

Secondly, we asked if there is evidence of a relationship between students’ personality and prior AA? Results indicate that AA was not significantly associated with any of the personality factor variables. As noted above, while the competitive and highly selective entry into teacher education is generally positively regarded UG pre-service teachers gained access to the program largely through prior AA on a standardized state run examination. This is therefore a notable finding, because it suggests that personality development among students is something that would need to be addressed within programs, rather than through selection based on prior AA in order to promote desirable adult development.

Thirdly, we asked if there is evidence of a relationship between students’ personality and social desirability. Results indicate evidence of a relationship between students’ social desirability and two personality scales: agreeableness and emotional stability. The relationship is positive (those students who score higher on social desirability tended to report higher initial agreeableness and emotional stability scores). The association is a moderate association which explains a good proportion of the variance in initial levels of personality. It is possible that the relationship between social desirability and personality is moderate because this outcome is influenced by many different factors, such as IQ, motivation, and the influence of parents and peers.

Prior research, particularly the conclusion that personality is not set in stone but instead continues to develop into adulthood, has implications for many important outcomes, including AA. Personality, most notably the trait of conscientiousness, is known to be related to academic success and educational attainment. Indeed, the association between conscientiousness and academic performance is nearly as strong as that between intelligence and academic performance. Studies have shown that interventions are effective in altering personality factors, and these may be used to enhance factors that have a positive effect on educational outcomes. It remains to be seen whether interventions designed to increase pre-service teachers’ personality factors might be one way to improve teaching and learning.

## Author Contributions

Study conception and design: RC. Acquisition of data: RC. Analysis and interpretation of data: RC. Drafting of manuscript: RC. Critical revision: RC. Acquisition of data: JF. Drafting of manuscript: JF.

## Conflict of Interest Statement

The authors declare that the research was conducted in the absence of any commercial or financial relationships that could be construed as a potential conflict of interest.
